# Myasthenia-Like Presentations Following PD-1 Inhibitors: CD8+ Myositis, Myasthenia, or Both?

**DOI:** 10.7759/cureus.103430

**Published:** 2026-02-11

**Authors:** Selen Ucem, Gulshan Yunisova, Eser Bulus, Sahin Lacin, Piraye Oflazer

**Affiliations:** 1 Department of Neurology, Koç University Hospital, İstanbul, TUR; 2 Muscle Diseases Center, Department of Neurology, Koç University Hospital, İstanbul, TUR; 3 Department of Oncology, Koç University Hospital, İstanbul, TUR

**Keywords:** immune checkpoint inhibitor, immune-related adverse effects, myasthenia, myositis, pembrolizumab, programmed cell death protein 1

## Abstract

Immune checkpoint inhibitors (ICIs) can trigger immune-mediated neuromuscular complications, where myositis and myasthenia frequently overlap, creating major diagnostic and therapeutic challenges.

We retrospectively analyzed five patients who developed acute neuromuscular symptoms after anti-programmed death-1 (PD-1) therapy. Clinical data, laboratory findings, electromyography, muscle biopsy results, treatments, and outcomes were reviewed.

All patients presented with varying combinations of oculobulbar weakness, dysphagia, limb weakness, and respiratory failure. Despite the myasthenia-like presentation, anti-acetylcholine receptor and anti-muscle-specific kinase antibodies were negative in four of five patients. In contrast, electromyography predominantly revealed subacute myogenic findings, with one patient exhibiting a neurogenic pattern. Muscle biopsies demonstrated CD8⁺-predominant inflammatory infiltration. The diagnosis of coexisting myasthenia was supported based on fluctuating symptoms and ventilator requirements, such as tidal volume and inspiratory pressure, as well as pyridostigmine responsiveness, rather than serologic findings. Most patients required immunosuppressive therapy, including corticosteroids, intravenous immunoglobulin, and, in some cases, cardiac or respiratory support.

ICI-related neuromuscular syndromes frequently present with myasthenia-like symptoms but are primarily driven by CD8⁺-mediated myositis rather than classical antibody-mediated pathology. Fluctuating respiratory or bulbar symptoms and response to pyridostigmine can guide differentiation between isolated myositis and coexisting myasthenia, which is crucial for optimizing ventilatory management. Therefore, a diagnostic trial of pyridostigmine is recommended in cases of ICI-related respiratory or bulbar deterioration, even in seronegative patients, as it may help clarify the underlying mechanism and guide timely therapeutic decisions.

## Introduction

Immune checkpoint inhibitors (ICIs) exert anti-tumoral activity by disinhibiting T-lymphocyte activation, most commonly through programmed cell death protein (PD-1), such as pembrolizumab and nivolumab, its ligand (PD-L1), cytotoxic T-lymphocyte-associated antigen-4 (CTLA-4), and lymphocyte activation gene 3 (LAG3). Although effective, they may trigger neurological immune-related adverse events (NirAEs) in 1%-6% of patients [1]. Among these, myasthenia, inflammatory myositis, and neuropathies are established but remain diagnostically challenging. Antibody seronegativity, overlapping features, and atypical presentations often delay recognition, while late intervention increases mortality. Overlap syndromes, particularly myasthenia with myositis, with or without myocarditis, further complicate management and urge prompt diagnosis and treatment [2]. We present five patients who developed neuromuscular complications following pembrolizumab/nivolumab. Highlighting seronegative myasthenia responsive to pyridostigmine, myositis with oculobulbar involvement and myocarditis, and acute demyelinating polyradiculopathy with coexisting myasthenia, we present these cases to underscore the value of early treatment, multidisciplinary collaboration, and proactive monitoring.

## Case presentation

Case 1

An 87-year-old man with metastatic lung adenocarcinoma developed progressive neck weakness, dyspnea, and fatigue one month after his second pembrolizumab cycle. He rapidly progressed to respiratory failure and bradycardia, requiring ventilation and a pacemaker. His neurological examination revealed bilateral ptosis, severe tongue and neck weakness, and mild generalized limb weakness with globally hypoactive deep tendon reflexes (DTRs). Lab tests were within normal limits (Table 1). A pyridostigmine trial (60 mg four times daily) improved neck flexion strength (Medical Research Council (MRC) Score 2/5 to 4/5). With the differential diagnosis of neuromuscular junction disorders in mind, anti-acetylcholine receptor (anti-AchR) antibody, anti-muscle-specific kinase (anti-MuSK) antibody, anti-titin antibody, and anti-voltage-gated calcium channel (VGCC) antibody tests were performed, all of which returned negative (Table 1). Electromyography (EMG) did not reveal any decremental response in the repetitive nerve stimulation study, while needle EMG demonstrated predominantly subacute myogenic changes with acute denervation potentials. The muscle biopsy confirmed inflammatory myositis with necrosis. He received intravenous immunoglobulin (IVIG) (0.4 g/kg/day for five days, with monthly maintenance) and oral prednisolone (30 mg/day). Within five weeks, he was gradually weaned from ventilation, regained bulbar and neck strength, and ambulated with support. The clinical picture was attributed to ICI-related myositis with coexisting seronegative myasthenia. Unfortunately, he died one year later due to the progression of his cancer.

**Table 1:** Clinical characteristics; biochemical, electrophysiological, imaging, and histopathology results; diagnosis; and prognosis of the five cases ICI: immune checkpoint inhibitor, CK: creatine kinase, anti-AchR: anti-acetylcholine receptor antibody, anti-MuSK: anti-muscle-specific kinase antibody, EMG: electromyography, MRI: magnetic resonance imaging, IVIG: intravenous immunoglobulin, IVMP: intravenous methylprednisolone MUAPs: motor unit action potentials.

Patient number	1	2	3	4	5
ICI treatment	Pembrolizumab	Pembrolizumab	Pembrolizumab	Nivolumab	Nivolumab
Onset	1 month after the second cure	15 days after the second cure	1 month after the second​​​​​​​ cure	3 weeks after the second​​​​​​​ cure	2 weeks after the second​​​​​​​ cure
Clinical Presentation	Weakness of neck flexion, fatigue, and progressive dyspnea	Bilateral ptosis, diplopia, and fatigue	Left arm weakness evolving into quadriparesis	Progressive difficulty in walking, dysphagia, and dyspnea	Difficulty in walking, dysphagia, and progressive dyspnea
CK (N: 20-200 U/L)	101 U/L	1843 U/L	508 U/L	1315 U/L	101 U/L
Antibodies	Anti-AchR < 0.25 nmol/L, anti-MuSK ​​​​​​​< 0.01 nmol/L, paraneoplastic antibody panel: negative autoimmune inflammatory myositis panel: Ro-52 (+)	Anti-AchR < 0.25 nmol/L, anti-MuSK ​​​​​​​< 0.01 nmol/L, paraneoplastic antibody panel: negative	Anti-AchR: 1.07 nmol/L (<0.25 nmol/L), anti-MuSK^4^ ​​​​​​​< 0.01 nmol/L, paraneoplastic antibody panel: negative	Anti-AchR < 0.25 nmol/L, anti-MuSK ​​​​​​​< 0.01 nmol/L, paraneoplastic antibody panel and autoimmune myositis: negative	Anti-AchR < 0.25 nmol/L, anti-MuSK ​​​​​​​< 0.01 nmol/L, paraneoplastic antibody panel: negative
EMG	Myogenic MUAPs with acute denervation potentials	Not performed	Neurogenic MUAPs, no F-wave responses	Myogenic MUAPs, acute denervation potentials	Myogenic MUAPs, acute denervation potentials
MRI	Not performed	Not performed	Contrast enhancement in cranial, spinal nerves, pia mater, cauda equina fibers	Not performed	Not performed
Muscle biopsy	Inflammatory myositis with necrosis	Inflammatory myositis with necrosis	Not performed	Not performed	Not performed
Diagnosis	Inflammatory myopathy and myasthenia overlap syndrome	Inflammatory myopathy and myasthenia overlap syndrome	Acute inflammatory neuropathy and myasthenia overlap syndrome	Inflammatory myopathy and myocarditis	Inflammatory myopathy and myasthenia overlap syndrome
Treatment	IVIG 0.4 mg/kg/day, oral prednisolone 30 mg/day, pyridostigmine 60 mg 4x1 tb/day	IVMP 500 mg/day every other day, oral prednisolone 40 mg/day, pyridostigmine 60 mg 5x1 tb/day	IVIG 0.4 mg/kg/day, oral prednisolone 60 mg/day, and pyridostigmine 60 mg 4x1 tb/day	Oral prednisolone 30 mg/day, pyridostigmine 60 mg 3x1 tb/day	Oral prednisolone 30 mg/day, pyridostigmine 60 mg 4x1 tb/day
Follow-up	Improved, later died of cancer progression	Improved, later died of cancer progression	Improved, later died of cancer progression	Improved	Improved

Case 2

**Figure 1:**
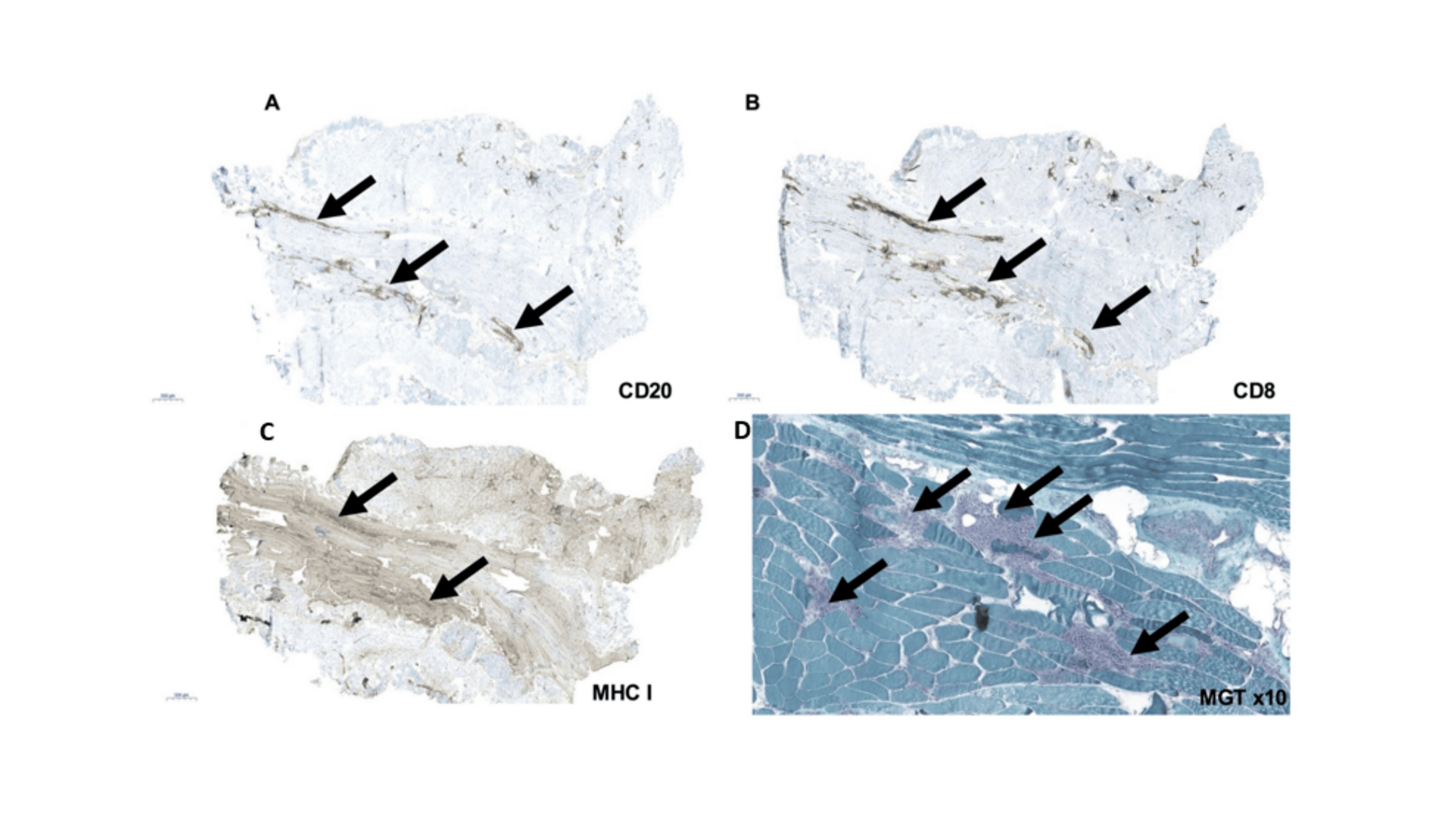
Muscle biopsy (A) B lymphocytes (CD20) (arrows) and (B) cytotoxic T lymphocyte (CD8) infiltrates (black arrows). (C) Patchy overexpression of major histocompatibility complex class I (MHC I), with relatively higher expression around areas of inflammatory cell infiltrates (arrows). (D) Modified Gomori trichrome (MGT) stain showing abundant perimysial and endomysial inflammatory infiltrates (black arrows).

A 79-year-old man with metastatic non-small cell lung carcinoma developed bilateral ptosis, diplopia, fatigue, and dysphagia 15 days after his second pembrolizumab cycle. He required supplemental oxygen and was unable to walk. His neurological examination revealed bilateral ptosis obscuring the pupils, restricted upward and lateral gaze, moderate weakness of the tongue, and globally hypoactive DTRs with preserved muscle strength. Serologic tests were normal (Table 1); creatine kinase (CK) was elevated (1843 U/L; N: 20-200). Muscle biopsy confirmed inflammatory myositis with necrosis (Figure 1). Given suspected overlap with myasthenia, an ice-pack test was performed and produced clear improvement in ptosis, providing supportive evidence for coexisting neuromuscular junction dysfunction. Furthermore, the pyridostigmine trial (60 mg twice daily) led to significant improvement in diplopia, ptosis, and dyspnea. Treatment included intravenous methylprednisolone (500 mg/day for five days), followed by oral prednisolone (40 mg/day) and pyridostigmine (60 mg five times daily). Within two weeks, he ambulated independently for 300 m and no longer experienced dyspnea or dysphagia. At one year, he remained ambulatory with mild ocular weakness, but died in the second year from progression of cancer.

Case 3

**Figure 2:**
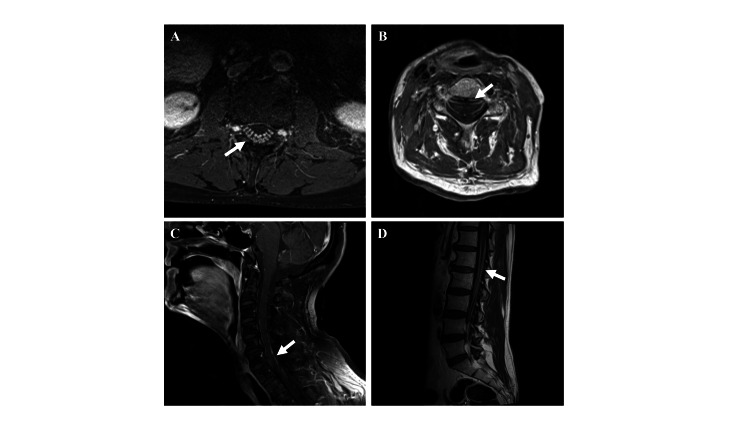
Diffuse spinal nerve root and pial enhancement on post-contrast MRI Post-contrast T1 images of (A, B) axial and (C, D) sagittal spinal cord showing contrast enhancement along the ventral and dorsal nerve roots of spinal nerves, cauda equina fibers, and pial surfaces throughout the spinal cord.

A 58-year-old man with laryngeal carcinoma developed acute left leg weakness progressing to quadriparesis within days, one month after his second pembrolizumab cycle. His neurological examination showed medial gaze palsy, orbicularis oculi weakness, facial paresis, severe tongue and neck flexion weakness, and profound limb weakness (MRC 1-2/5), with absent DTRs. Magnetic resonance imaging (MRI) demonstrated contrast enhancement of cranial nerves III, VI, and IX-XI and both ventral and dorsal spinal roots (Figure 2). Cerebrospinal fluid (CSF) analysis revealed albuminocytologic dissociation (CSF protein 396.6 mg/dL, 0 cells/µL; N: protein 15-45 mg/dL, 0 cells/µL). EMG showed absent F-waves with giant motor units and reduced recruitment, consistent with acute demyelinating polyradiculopathy. Despite IVIG (0.4g/kg/day for five days), he developed acute respiratory failure and hypotension, requiring invasive ventilation and vasopressors. Persistent ventilator dependence with fluctuating settings raised suspicion of neuromuscular junction dysfunction. Pyridostigmine (60 mg four times daily) improved tidal volume and reduced inspiratory pressure needs. Anti-AchR antibody was positive (1.07 nmol/L; normal < 0.25), and CK was mildly elevated (508 U/L). A diagnosis of coexisting myasthenia was made. Prednisolone (60 mg/day) and pyridostigmine were continued, leading to successful weaning from ventilation after three weeks, with marked bulbar and limb recovery (MRC 3-4/5). At one year, he was ambulatory with support and tolerated oral feeding; however, he later died from the progression of his cancer.

Case 4

An 83-year-old man with pleural mesothelioma developed progressive gait difficulty, dysphagia, and dyspnea three weeks after his second nivolumab cycle. He sustained respiratory arrest requiring resuscitation and tracheostomy. Examination showed ptosis, moderate tongue weakness, severe neck flexor weakness, and symmetric proximal limb weakness. Serologic tests were negative (Table 1), CK was elevated (1315 U/L; N <190), and troponin-T was markedly increased (1370 ng/L; N <24.9). Echocardiography revealed regional wall motion abnormalities, consistent with myocarditis secondary to myositis. EMG demonstrated myogenic motor unit action potentials (MUAPs) with acute denervation in proximal muscles. A pyridostigmine trial showed no clinical benefit. Hence, ICI-induced inflammatory myositis was diagnosed, and oral prednisolone (30 mg/day) was started. Over three weeks, muscle strength improved to 4+/5 in the extremities, and tracheostomy was closed. At the six-month follow-up, he remained off ventilation and maintained oral intake.

Case 5

A 66-year-old woman with gastric adenocarcinoma developed progressive gait difficulty, dysphagia, and dyspnea one month after her fifth nivolumab dose. Her course was complicated by respiratory failure requiring intubation. Thoracoabdominal imaging excluded embolism and infection. Examination showed ptosis, gaze paresis, severe neck flexor weakness, and moderate generalized weakness. A pyridostigmine trial improved the negative inspiratory force by 40%. Lab tests were within normal limits (Table 1). EMG showed myogenic MUAPs with denervation. Repetitive EMG was normal. Based on clinical/electrophysiological findings, ICI-related myositis with myasthenia was diagnosed. She was treated with oral prednisolone (30 mg/day) and pyridostigmine (60 mg four times daily). The patient was extubated on day 4 and achieved ambulation within two months. At the six-month follow-up, she remained neurologically stable without cancer recurrence.

## Discussion

With the increasing use of ICIs, the spectrum of NirAEs continues to expand. Unlike classical myositis, which primarily affects proximal limb muscles, ICI-induced myositis often involves extraocular, bulbar, and respiratory muscles, thereby mimicking or overlapping with myasthenia. Recent cohort studies indicate that ICI-related myositis is more frequent than ICI-induced myasthenia, which appears to coexist with myositis rather than an isolated entity. Plomp et al. showed florid CD8+ T cell and CD68+ macrophage infıltrates in the extraocular muscles and diaphragm of ICI-related myositis/myasthenia cases, explaining the oculobulbar and respiratory symptoms [2]. The underlying mechanism appears to involve the loss of peripheral immune tolerance under PD-1/PD-L1 blockade, driving uncontrolled CD8⁺/CD4⁺ T cells, macrophages, and B-cell activity [3]. Therefore, it is legitimate to conceive that an uncontrolled immune response may involve both muscle and the neuromuscular junction, reflecting a distinct immunopathogenic process of myasthenia (a neuromuscular junction dysfunction) rather than classical antibody-mediated disease of MG [2,3]. Thus, in our patients, the MG-like picture may represent a bystander effect of intense CD8⁺ T-cell-mediated inflammation at the neuromuscular junction. Plomp et al. detected AchR antibodies only in a minority of cases, yet these patients did not exhibit fatigable or fluctuating weakness, decrement on repetitive nerve stimulation, or response to pyridostigmine, as in Case 3; in some, AchR antibodies even predated the ICI therapy [2]. In vitro studies by Masi et al. further demonstrate that these AchR antibodies lack pathogenic effector functions, implicating alternative immune mechanisms in myasthenia pathogenesis [4]. Conversely, a seronegative patient may still be diagnosed with myasthenia if clinical evidence is strong. Indeed, up to 30%-50% of ICI-related myasthenia cases are seronegative. Similar to our series, in which four of five patients were seronegative, this highlights the importance of clinical assessment [5]. In our series, fluctuating features, such as variable ventilator settings, activity-induced fatiguability, and episodic ptosis or MRC score fluctuations, supported neuromuscular junction involvement even in the absence of antibodies. Importantly, a positive pyridostigmine response should not be interpreted as diagnostic of myasthenia gravis (MG); rather, it serves as supportive evidence of neuromuscular junction dysfunction, guiding acute management but not defining classical MG. Since respiratory failure is a frequent, life-threatening complication of ICI-related neuromuscular disorders, even in seronegative cases, a pyridostigmine trial alongside early immunosuppression may both confirm neuromuscular junction involvement and improve outcomes [6]. Furthermore, cardiovascular complications, particularly myocarditis, are observed in a significant subset of patients. Routine screening with troponin levels and electrocardiographic monitoring is recommended to ensure timely detection and management of these fatal events [7].

Clinical implications

In patients receiving PD-1 inhibitors who developed fluctuating bulbar, ocular, or respiratory symptoms, clinicians should maintain a high suspicion for ICI-induced myositis with potentially coexisting MG-like neuromuscular junction dysfunction. A short therapeutic trial of pyridostigmine may provide supportive evidence of junctional involvement and help guide acute management, although it should not be interpreted as diagnostic of MG.

Given the substantial risk of concomitant myocarditis in this population, routine screening with troponin measurements and electrocardiographic monitoring is recommended to allow early identification and timely treatment of potentially life-threatening cardiac complications.

## Conclusions

ICI-induced neuromuscular disorders require a comprehensive approach integrating clinical, electrophysiological, serological, and histopathological findings. Atypical muscle involvement (particularly oculobulbar or respiratory) should raise the suspicion for ICI-related myositis with possible coexisting neuromuscular junction dysfunction. In such cases, a carefully monitored pyridostigmine trial may assist diagnosis in unexplained respiratory failure. Early recognition of these features enables timely intervention that can avert fatal outcomes. Therefore, a diagnostic trial of pyridostigmine is recommended in cases of suspected ICI-related respiratory failure, even in seronegative patients, to guide management. The phenotypic overlap between ICI-related myositis and myasthenia presentations likely reflects widespread CD8⁺ T-cell-mediated inflammation, with clinical manifestations determined by the specific muscle groups affected. Further prospective studies are needed to validate these observations and better define the pathomechanism of neuromuscular junction involvement and the mechanistic interplay between myositis and neuromuscular junction dysfunction in ICI-treated patients.
